# Synergy-Based Evaluation of Hand Motor Function in Object Handling Using Virtual and Mixed Realities

**DOI:** 10.3390/s25072080

**Published:** 2025-03-26

**Authors:** Yuhei Sorimachi, Hiroki Akaida, Kyo Kutsuzawa, Dai Owaki, Mitsuhiro Hayashibe

**Affiliations:** 1 Neuro-Robotics Lab, Department of Robotics, Graduate School of Engineering, Tohoku University, Sendai 980-8579, Japan; yuhei.sorimachi.q1@dc.tohoku.ac.jp (Y.S.);; 2 Department of Biomedical Engineering, Graduate School of Biomedical Engineering, Tohoku University, Sendai 980-8579, Japan

**Keywords:** synergy, hand motor function, leap motion controller, mixed reality, HoloLens 2

## Abstract

This study introduces a novel system for evaluating hand motor function through synergy-based analysis during object manipulation in virtual and mixed-reality environments. Conventional assessments of hand function are often subjective, relying on visual observation by therapists or patient-reported outcomes. To address these limitations, we developed a system that utilizes the leap motion controller (LMC) to capture finger motion data without the constraints of glove-type devices. Spatial synergies were extracted using principal component analysis (PCA) and Varimax rotation, providing insights into finger motor coordination with the sparse decomposition. Additionally, we incorporated the HoloLens 2 to create a mixed-reality object manipulation task that enhances spatial awareness for the user, improving natural interaction with virtual objects. Our results demonstrate that synergy-based analysis allows for the systematic detection of hand movement abnormalities that are not captured through traditional task performance metrics. This system demonstrates promise in advancing rehabilitation by enabling more objective and detailed evaluations of finger motor function, facilitating personalized therapy, and potentially contributing to the early detection of motor impairments in the future.

## 1. Introduction

In recent years, advancements in medical technology, along with global economic growth, have contributed to a rapidly aging population worldwide [1]. As individuals age, it is well documented that both motor and cognitive functions decline, increasing the risk of incidents such as falls [2,3]. Consequently, the demand for rehabilitation services aimed at mitigating these risks is expected to rise in parallel with the growing elderly population. Rehabilitation is crucial for preventing the need for long-term care, alleviating symptoms, and maintaining mobility. However, current assessments of motor function are often subjective, heavily relying on the therapist’s experience and visual judgments. This underscores the increasing demand for automated, quantitative systems capable of objectively evaluating motor function during rehabilitation.

Among human motor functions, finger movements are particularly essential. Fingers are capable of highly dexterous actions, enabling tasks such as touching objects, carrying objects, and expressing emotions. Therefore, maintaining finger function is essential for preventing a decline in quality of life (QoL). Furthermore, it is well known that finger movements are closely related to brain function [4,5,6]. Given the significance of finger function and its connection to the brain, there is a pressing need for automated, quantitative methods to assess finger function in rehabilitation. Methods for assessing upper extremity motor function, including finger movements, include the Barthel Index (BI) [7], Disabilities of Arm, Shoulder and Hand (DASH) [8], Fugl–Meyer Assessment (FMA) [9], and Wolf Motor Function Test (WMFT) [10]. These assessments primarily evaluate activities of daily living (ADLs) such as eating, dressing, and bathing. While these assessment methods focus on finger performance, they often involve therapists visually scoring the patient’s movements or relying on patient-reported outcomes, where patients answer a series of questions to determine their motor function status. Although some studies have investigated the quantitative assessment of hand motor function, they are limited to analyzing the range of motion of the wrist joint [11,12] and examining grasp trajectory and velocity [13,14].

High-degree-of-freedom motor functions, which involve the movement of multiple joints and muscles, contain a wealth of multidimensional information. To analyze such complex data, many studies employ dimensionality reduction techniques like principal component analysis (PCA) and nonnegative matrix factorization (NMF) to extract joint and muscle coordination structures (patterns) [15,16,17,18,19,20,21,22,23,24,25]. These concepts of kinematic and dynamic coordination are commonly referred to as the synergy hypothesis. Lee et al. [26] utilized synergy to assess the ability of healthy subjects and stroke patients to control finger force. Jarque et al. [27] identified spatial synergy in 26 representative ADLs among 22 healthy subjects. Camardella et al. [28] analyzed the correlation between muscle synergy in both healthy subjects and stroke patients, as well as the relationship between changes in muscle synergy [29] and alterations in clinical scale scores following rehabilitation. Thus, evaluating motor function from the perspective of synergy can elucidate the differences in motor abilities between healthy and disabled individuals and can aid in developing training methods to enhance hand function.

Rehabilitation generally involves the repetition of monotonous movements over extended periods, which can make it challenging to keep patients motivated. Consequently, research on the gamification of rehabilitation is underway [30,31,32]. Gamification is defined as “the use of game-specific designs and elements in non-game contexts” [33]. Incorporating game elements into repetitive rehabilitation exercises has been shown to enhance patient motivation and attention [34,35]. Virtual reality (VR) training is a technology that combines motor function assessment with gamification. VR replaces the subject’s surrounding environment with a computer-generated virtual space, allowing individuals to train in an immersive and game-like manner, which can lead to increased motivation. Additionally, there are technologies such as augmented reality (AR), which adds virtual objects to the real environment, and mixed reality (MR), which integrates the real and virtual worlds. Several studies have applied these technologies to rehabilitation [36,37,38,39,40,41].

Meanwhile, traditional glove-type devices have been used to evaluate finger movements in detail [26]; however, these devices often restrict joint motion and prove challenging for patients with severely deformed fingers, such as those affected by stroke spasticity. To address the challenges identified in previous research, this study leverages VR and MR technologies, which allow for the analysis of finger kinematics without the constraints imposed by conventional instruments. By integrating synergies, this approach enables a comprehensive evaluation of finger motor functions and features, offering insights that task performance alone cannot capture.

The purpose of this study is to evaluate finger motor function from a synergy perspective through a training game designed for manipulating a virtual object by using pervasive devices. We developed this training game utilizing the leap motion controller (LMC) (Leap Motion, Inc., San Francisco, CA, USA), a markerless motion sensor. Spatial synergies were extracted from finger motion data during the grasping operation. This process was conducted on multiple healthy subjects to establish a standard model of synergy combinations. Using this standard synergy as a reference, we assessed the finger function of both healthy and simulated disabled subjects. Additionally, we developed an MR virtual object manipulation game using HoloLens 2 (Microsoft, Redmond, WA, USA), a head-mounted device, to enhance the training efforts while keeping a realistic sense of the environment.

## 2. Materials and Methods

### 2.1. Synergy Extraction

#### 2.1.1. Spatial Synergy

In this study, finger movements are analyzed using spatial synergy, which represents spatial coordination. Spatial synergy is extracted as a non-time-varying spatial coordination structure that captures low-dimensional structures hidden in high-dimensional time series data. Spatial synergy is represented by *w* in the following equation.

(1) $$j\left(t\right)\approx \sum_{i=1}^{N}c_{i}\left(t\right)w_{i}+\bar{j},\;N\leq \dim j\left(t\right)$$

Here, j(t) represents the joint angles at time *t*, *t* represents time, *i* represents the synergy number, *N* represents the total number of synergies, $c_{i}\left(t\right)$ represents the time-varying coefficient indicating the intensity of i-th synergy at time *t*, $w_{i}$ represents the i-th synergy, and $\bar{j}$ represents the average joint angle. The synergy $w_{i}$ contains information about which joints move in coordination with each other, while $c_{i}\left(t\right)$ contains information about the intensity and timing of the usage of each synergy. Assuming that there is a correlation between coordinated movements and motor function, it is believed that using synergies facilitates the analysis and evaluation of movement data. This study uses principal component analysis (PCA), which is widely used for low-dimensional joint motion by human grasping, and a method that combines PCA and varimax rotation. These methods are described in a later section.

#### 2.1.2. Cumulative Contribution Ratio and Eigenvalue

The synergy extracted by spatial synergy and how much the intensity and timing of its use contribute to the original data is measured by the variance accounted for (VAF). VAF is defined as follows [42]:

(2) $$VAF=1-\frac{\sum_{Joint}{∥x-x_{N}∥}^{2}}{\sum_{Joint}{∥x∥}^{2}}$$

x represents the original joint angle data, and $x_{N}$ represents the data reconstructed using *N* synergies. The closer the value of VAF is to 1, the closer the approximation by *N* synergies is to the original data. In PCA, the eigenvalues correspond to the magnitude of variance explained by each synergy. In this study, the criterion for selecting the number of synergies was to choose principal components with eigenvalues greater than one. This method has been widely used in previous studies on finger synergies [15,27].

#### 2.1.3. Principal Component Analysis (PCA)

PCA is a dimensionality reduction technique that approximates correlated joint movement data using a low-rank matrix *W*, consisting of *N* linearly independent basis vectors called principal components (PCs) $w_{i}$, and a time-varying coefficient matrix *C* with row vectors representing the coefficients $c_{i}$ of each basis vector Equation (1). When transforming into PCs, the variance of the first PC is maximized initially. The subsequent principal components are determined to maximize variance under the condition that they are orthogonal to other PCs. A characteristic feature of synergies obtained through PCA is that the first PC accounts for most of the variance in the original data.

#### 2.1.4. Varimax Rotation

Varimax rotation is an orthogonal rotation method proposed by Kaiser et al. [43]. In varimax rotation, the PCs are rotated so that the factors within the PCs are maximized or close to zero while maintaining their orthogonality. Synergies that contain only a few factors with high loadings on each variable are called sparse synergies. Synergies used in human control are considered to have this sparse property. Therefore, synergies with sparse properties are considered suitable for human body motion analysis. In this study, we apply the varimax rotation to the synergies extracted by PCA. Varimax rotation *R* is a N×N orthogonal matrix that maximizes the following function.

(3) $$v\left(R,W\right)=\frac{1}{N_{joint}}\sum_{j=1}^{N}\left\{\sum_{i=1}^{N_{joimt}}{{\left[WR\right]}_{ij}}^{4}-\frac{1}{N_{joint}}{\left(\sum_{i=1}^{N_{joint}}{{\left[WR\right]}_{ij}}^{2}\right)}^{2}\right\}$$

$N_{joint}$ represents the total number of joint angles. W represents a $N\times N_{joint}$ synergy matrix, which compiles all *N* synergies. Specifically, $W=\left[w_{1},w_{2},\ldots ,w_{N}\right]$. The rotation matrix $R^{*}$ that maximizes the above function is applied to the synergy obtained by PCA to obtain a more sparse synergy matrix $W^{\prime }$ and synergy activation patterns $C^{\prime }$ by the following formula.

(4) $$\begin{array}{cc} J & =WC+\bar{J}\\ \; & =WR^{*}\;{R^{*}}^{T}C+\bar{J}\\ \; & =W^{\prime }C^{\prime }+\bar{J} \end{array}$$

J represents a matrix of joint angles, which compiles the joint angle vectors at all times *t*. Specifically, $J=\left[j\left(t_{1}\right),j\left(t_{2}\right),\ldots ,j\left(t_{f}\right)\right]$. C represents a matrix of synergy usage, which compiles the time-varying coefficient indicating the intensity of synergy at each time *t*. Specifically, C is expressed by the following equation.

(5) $$C=\left[\begin{array}{cccc} c_{1}\left(t_{1}\right) & c_{1}\left(t_{2}\right) & \cdots & c_{1}\left(t_{f}\right)\\ c_{2}\left(t_{1}\right) & c_{2}\left(t_{2}\right) & \cdots & c_{2}\left(t_{f}\right)\\ \vdots & \vdots & \ddots & \vdots \\ c_{N}\left(t_{1}\right) & c_{N}\left(t_{2}\right) & \cdots & c_{N}\left(t_{f}\right) \end{array}\right]$$

### 2.2. Virtual Object Manipulation Game

#### 2.2.1. Leap Motion Controller and VR Game Content

**Figure 1:**
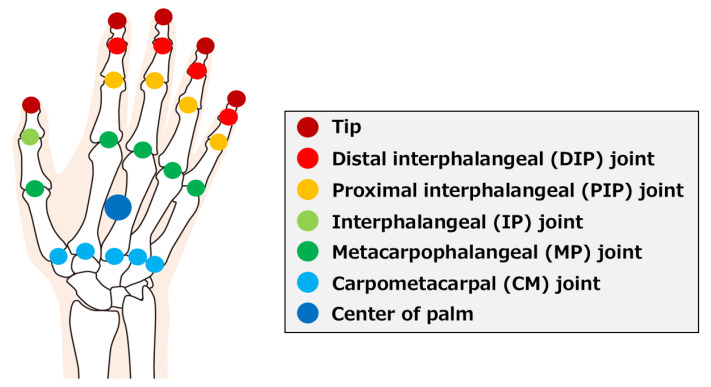
Coordinate positions obtained by LMC. A total of 25 coordinates were obtained for the center of the palm, the tip of each finger, each distal interphalangeal (DIP) joint, each proximal interphalangeal (PIP) joint, each metacarpophalangeal (MP) joint, interphalangeal (IP) joint, and each carpalmetacarpal (CM) joint.

In this study, we developed a virtual object manipulation game using Ultraleap’s leap motion controller (LMC) [44], a markerless motion sensor (Movie S1). The LMC is composed of two infrared cameras and three infrared-illuminated LEDs, and the spatial coordinates of the hand joints are obtained by measuring the time that the LED light travels between the sensor and the object. The spatial coordinates obtained by the LMC are shown in Figure 1.

**Figure 2:**
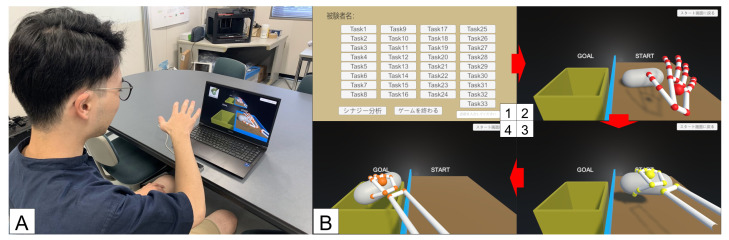
Leap Motion Controller (LMC) virtual object manipulation game. (**A**) PC and LMC are connected and operated by moving the hand above the LMC. (**B**) Game screen. 1: Task selection screen. 2: Virtual object appears on the right side. 3: Grasping the virtual object. 4: Carrying the virtual object to the goal on the left side.

Figure 2 shows the game in progress and the game screen. Unity was used to develop the game. By moving their own hands, the participants manipulate the virtual hand displayed on the screen to grasp an object and carry it to the goal. If the object falls, the task fails, and the subject tries the task again until it succeeds. Virtual object manipulation is performed using thirty-three different grasping methods proposed by Feix et al. [45]. After each grasping task, time series data of the hand joint angles are acquired. Synergies are extracted from the obtained joint angle data.

#### 2.2.2. Data Acquisition and Preprocessing

In this study, joint angle data from 18 joints (IP joint, MP joint, PIP joint, DIP joint, and adduction and abduction) were used for synergy extraction. These joint angles were calculated using the inner product after obtaining the joint coordinates shown in Figure 1. The joint angle data includes the state in which the hand is not moving and the state in which the arm is moving while maintaining the grasping posture. Therefore, we extracted the data during the grasping motion. The number of data frames differed because the time required for the grasping motion differs among grasping methods and individuals. Therefore, resampling was applied to normalize the time of the obtained data. Cubic spline interpolation was applied to the time series data for each joint angle, and resampling was applied by dividing the data into 20 equally sized frames.

### 2.3. Mixed Reality Object Manipulation Game

#### HoloLens 2 and MR Game Content

In order to enhance the usability of the game, this study also developed a virtual object manipulation game using Microsoft’s HoloLens 2 [46] MR head-mounted display (Movie S2). In the system using LMC, it was necessary to move the hand above the LMC while looking at the virtual hand displayed on the PC screen. In addition, because the VR space is displayed on the PC screen, it was sometimes difficult to grasp a sense of distance, especially when grasping thin objects. We used MR to solve these problems. HoloLens 2 projects images onto a see-through lens, which is overlaid on the real field of view to make virtual objects appear as if they exist in the real field. HoloLens 2 is equipped with an infrared sensor and an acceleration sensor, enabling it to acquire information on the surrounding environment and position and to interact with real-world objects. HoloLens 2 can acquire hand joint position information, as shown in Figure 1, like LMC.

**Figure 3:**
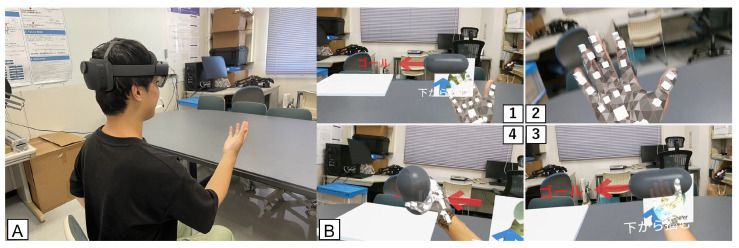
HoloLens 2 object manipulation game. (**A**) Wear HoloLens 2 and perform the task while sitting on the chair. (**B**) Game screen. 1: A virtual object is displayed on the right side, and the player grabs and carries the object to the whiteboard (goal) on the left side. 2: A virtual hand appears overlaid on the player’s hand. 3: Grabbing the virtual object. 4: The virtual object is carried to the goal on the left side.

Figure 3 shows the game in progress and the game screen. The game was developed using Unity and the Mixed Reality Toolkit (MRTK), a cross-platform development toolkit. The subject manipulates a virtual object displayed through a lens by moving a virtual hand that is overlaid on his or her own hand. The type of grasping method is the same as in the LMC version, but the subject is required to grasp the object from underneath. This is to suppress the effect of occlusion by the user’s own hand during the grasping motion. The HoloLens 2 has a sensor in the head, so the object is grasped from below so that the palm side of the hand can be measured. The data processing is the same as in LMC.

### 2.4. Experimental Design

This study consisted of three main experiments. In the first experiment, we extracted synergies in virtual object grasping in healthy subjects. We compared the synergies extracted by PCA and the method combining PCA and varimax rotation. In the second experiment, we verified whether synergy could be used to detect abnormal areas. We compared synergy between normal conditions and when the finger was fixed with tape. In Experiment 3, we developed an object manipulation task using an MR device and extracted synergy during virtual object grasping. A detailed description of each experiment is given below.

The study was conducted according to the guidelines of the Declaration of Helsinki and was approved by the ethical committee of Tohoku University under 22A-27. The experimental protocol and the study purpose were explained to all subjects before obtaining informed consent and starting the experiment.

#### 2.4.1. Method in Experiment 1

Twelve right-handed male subjects with an average age of 23 years and no abnormal finger function carried out the LMC task to extract synergies during virtual object grasping. Subjects carried out the tasks on a PC with the LMC placed on a table in a seated position. All tasks in the game were instructed to be carried out with the right hand. Each task was carried out twice in a random order. After all tasks were completed, spatial synergies during virtual object grasping were extracted using PCA and PCA with varimax rotation. Hierarchical cluster analysis was applied to the synergies obtained from all subjects, and they were classified based on similarity. *N* groups were selected from the highest number of synergies belonging to a cluster. Here, *N* is the chosen number of synergies. The average of the synergies belonging to each cluster was calculated and used as the representative synergy of the healthy subjects in thirty-three different virtual object grasps.

#### 2.4.2. Method in Experiment 2

As a preliminary step in Experiment 2, ten different grasps were selected from thirty-three different grasps. The reason for selecting ten types was based on traditional methods of hand function assessment [7,10], where ten to seventeen tasks are used. A hierarchical clustering analysis was applied to the intensity and timing of spatial synergy use obtained in Experiment 1, and the thirty-three grasp types were classified into ten clusters based on similar synergy use patterns. The tasks used in Experiment 2 were randomly selected from each of these ten clusters.

**Figure 4:**
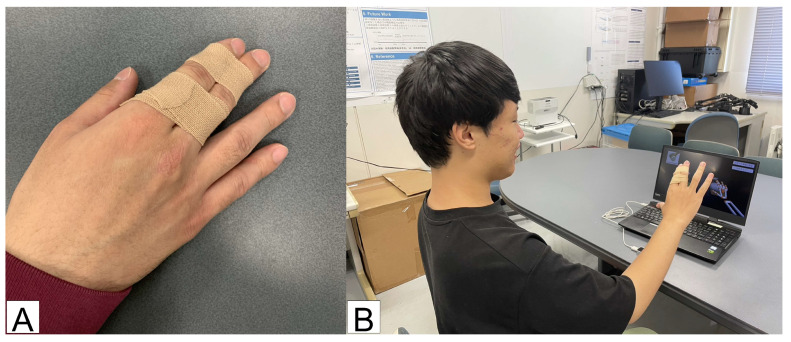
Condition of pseudo disability. (**A**) Fix index and middle fingers with tape. (**B**) Performing the task with the fingers fixed with tape.

In Experiment 2, subjects were asked to complete the task of moving a virtual object to a target location three times. If the virtual object is dropped at a location other than the start or goal location, it is considered a failure. The game consists of ten different selected tasks. All subjects play the game under two conditions: one with the index and middle fingers taped together to simulate a pseudo-obstacle, as shown in Figure 4, and one without restrictions. To assess hand function from the task performance, the time taken to complete the task three times and the success rate are measured. The following deviation equation is used as a measure of completion time and success rate.

(6) $$score=50-\frac{10(x-\mu )}{\sigma }$$

Similarity [47] and correlation are calculated to evaluate motor function in terms of synergy. The similarity is based on the cosine similarity obtained by the following equation. s1 and s2 represent the synergies to be compared, respectively. *i* is the number of synergies, and *N* is the total number of synergies.

(7) $$sim=\frac{1}{N}\sum_{i=1}^{N}\frac{∥s_{1i}\cdot s_{2i}∥}{∥s_{1i}∥∥s_{2i}∥}$$

Correlation is a value that expresses the degree of coordinated motion between two specific joints and is evaluated on a scale from −1 to 1. The closer the value is to 1, the stronger the interaction between the two joints. The closer it is to 0, the weaker or non-existent the coordination. The closer the value is to −1, the stronger the backward interaction. The equation for calculating the correlation is shown below.

(8) $$L_{J_{ij}}=\sum_{k=1}^{N}W_{k_{J_{i}}}\cdot W_{k_{J_{j}}}$$

$J_{i}$ and $J_{j}$ represent joints *i* and *j*, $w_{k_{J_{i}}}$ and $w_{k_{J_{j}}}$ represent the values of $J_{i}$ and $J_{j}$ of the *k*-th synergy, and *N* represents the total number of synergies. By calculating the difference in correlation between two joints, anomalies in joint coordination are detected.

#### 2.4.3. Method in Experiment 3

In Experiment 3, we extracted synergies during the grasping motion in the MR object manipulation game by using Hololens 2 to verify synergy extraction feasibility under MR training. The same ten grasping methods as in LMC were used, and each grasping method was performed three times. Synergies were extracted and compared to synergies obtained with LMC using synergy similarity. To assess the ease of playing the game in MR, the completion time of the game and the number of failures were also compared between VR and MR.

## 3. Results

### 3.1. Experiment 1

**Figure 5:**
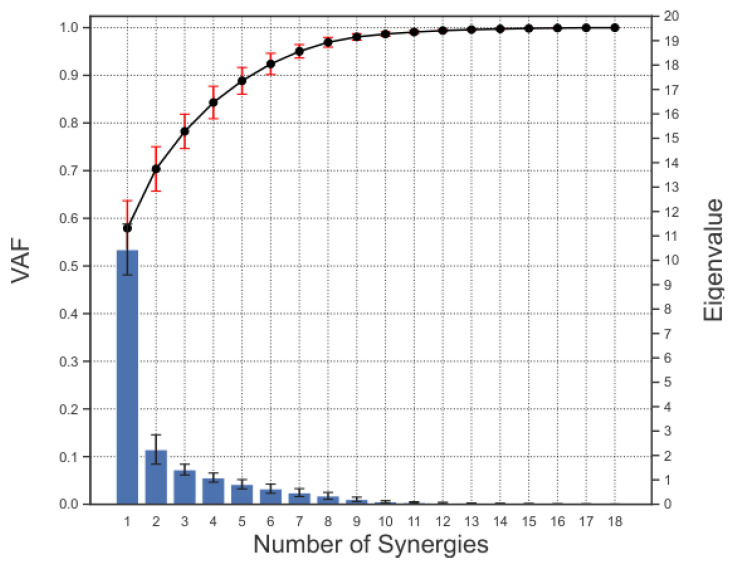
VAF and eigenvalues of synergy. Bars indicate eigenvalues for each synergy. Line graphs indicate VAF.

**Figure 6:**
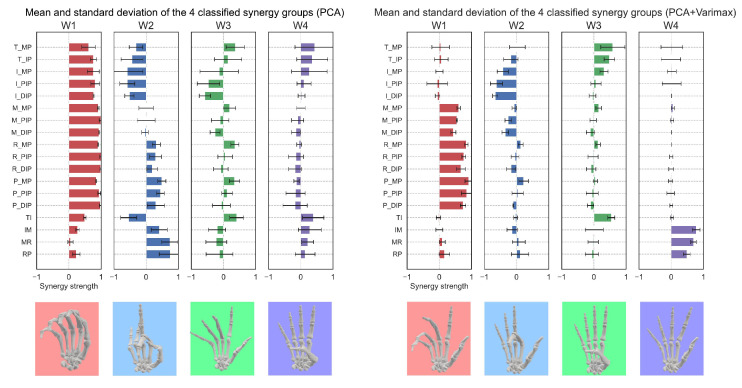
Comparison of PCA and PCA + Varimax synergies: (**Left**) PCA, (**Right**) PCA + Varimax. The images at the bottom depict corresponding finger movements in each synergy.

Figure 5 shows the VAF and eigenvalue results for each number of synergies of the spatial synergy obtained from the 12 subjects. Since the eigenvalues were greater than 1 up to the fourth synergy, four synergies were selected to be extracted. Hierarchical cluster analysis was performed on the 48 synergies obtained for all subjects (12 subjects × 4 synergies), and they were classified into 4 groups and a group consisting of a small number of synergies. Figure 6 (left) demonstrates the results of taking the mean and standard deviation of the synergies for each classified group. The video shows the hand movements in each synergy (sensors-25-02080).

Varimax rotation was applied to the four spatial synergies obtained by PCA. Hierarchical cluster analysis was applied, as in the case of synergies by PCA, and the results were classified into four groups and a group consisting of a small number of synergies. Figure 6 (right) shows the results of taking the mean and standard deviation of the synergies for each classified group by applying varimax rotation. The video shows the hand movements in each synergy (Movie S3). In PCA, synergies involving the movement of many joints are obtained. PCA with varimax rotation obtained synergies involving a small number of joints working together compared to PCA, indicating that a sparser synergy could have been obtained.

### 3.2. Experiment 2

**Figure 7:**
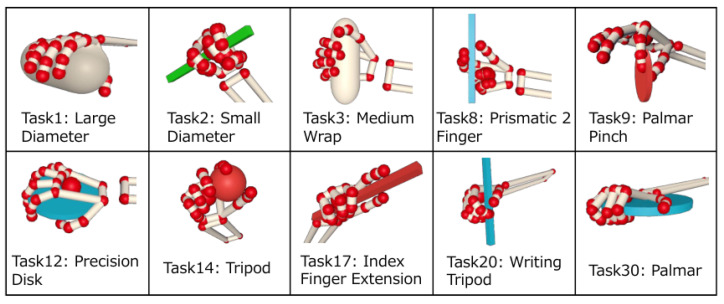
Ten different grasping methods chosen for the tasks in Experiments 2 and 3.

**Figure 8:**
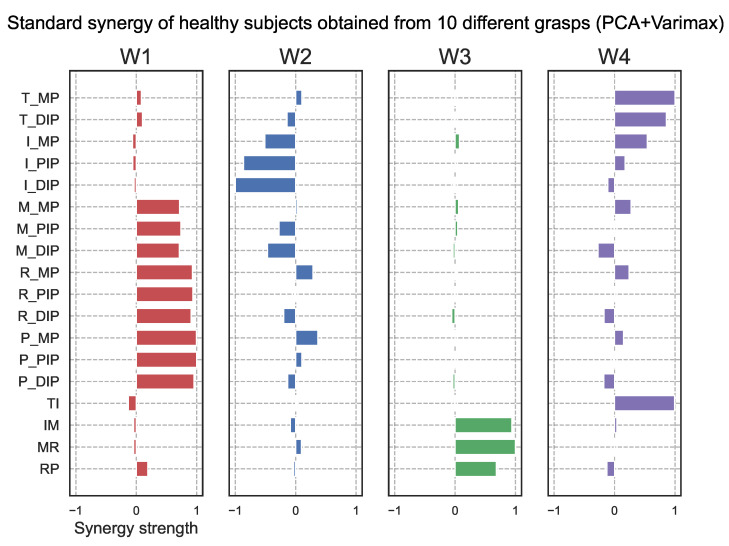
Standard synergies obtained from ten different virtual objects grasp data using PCA with varimax rotation.

Using the method described in the methodology, thirty-three different grasps [45] were categorized by similar actions and then narrowed down to ten different grasps. The selected tasks were Task1, Task2, Task3, Task8, Task9, Task12, Task14, Task17, Task20, and Task30 shown in Figure 7. Figure 8 shows the results of creating standard synergies for the ten grasping tasks for the healthy subjects based on the data obtained from Experiment 1. Each synergy is normalized so that the maximum absolute value is 1. Similar synergies were obtained from the ten task data as in the thirty-three task case. This synergy was used as the standard synergy for the ten healthy hand manipulations.

**Figure 9:**
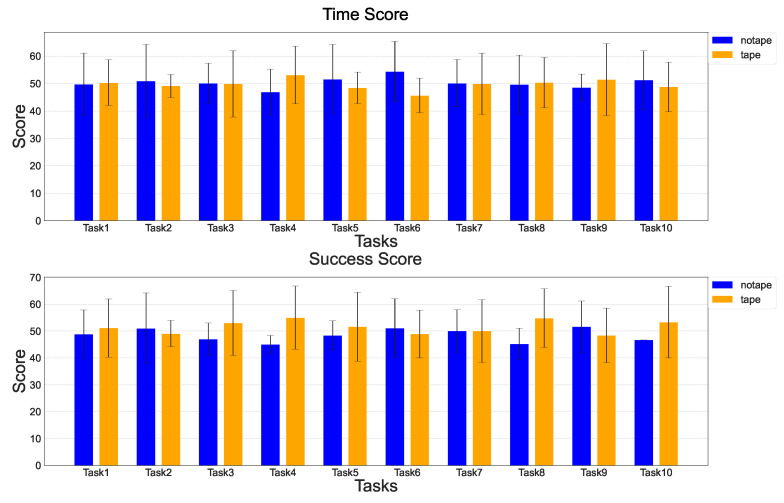
Task completion time and task success rate scores for each subject during normal (blue) and tape fixation (yellow) conditions.

In Experiment 2, subjects played the game in two ways: with nothing attached to their fingers and with the index and middle fingers fixed with tape. Time to completion and success rate were measured as indexes for evaluating hand function from a performance perspective. Figure 9 shows the mean value and standard deviation of the subjects’ deviation scores for each task.

**Figure 10:**
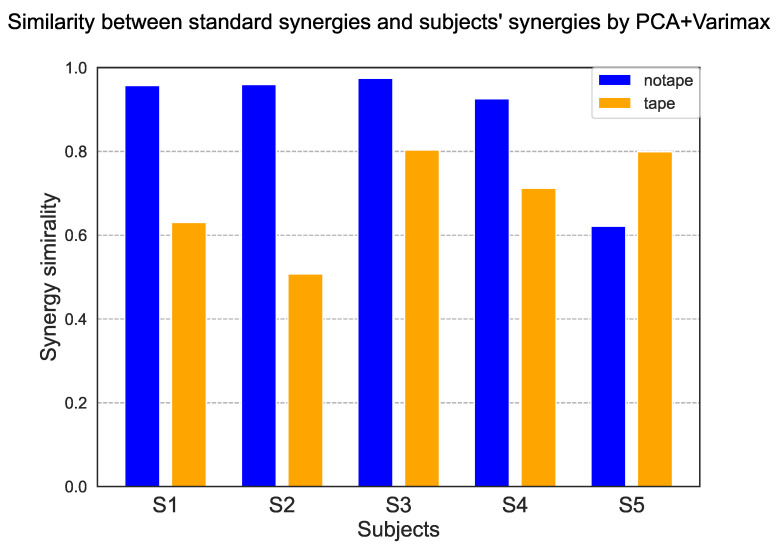
Similarity between the synergy obtained for each subject under normal conditions (blue) and under tape fixation (yellow) and the standard synergy.

To evaluate hand function in terms of synergy, we calculated the similarity between the synergy of each subject and the standard synergy of a healthy subject, as shown in Figure 8. Figure 10 shows the results of the synergy similarity for each subject. The graph shows that the similarity of all subjects, except subject 5, was higher for the case without tape fixation than for the one with tape fixation. The mean value without tape was 0.888 ± 0.150, and with tape was 0.691 ± 0.125. A *t*-test was performed, and *p* = 0.053 was obtained.

**Figure 11:**
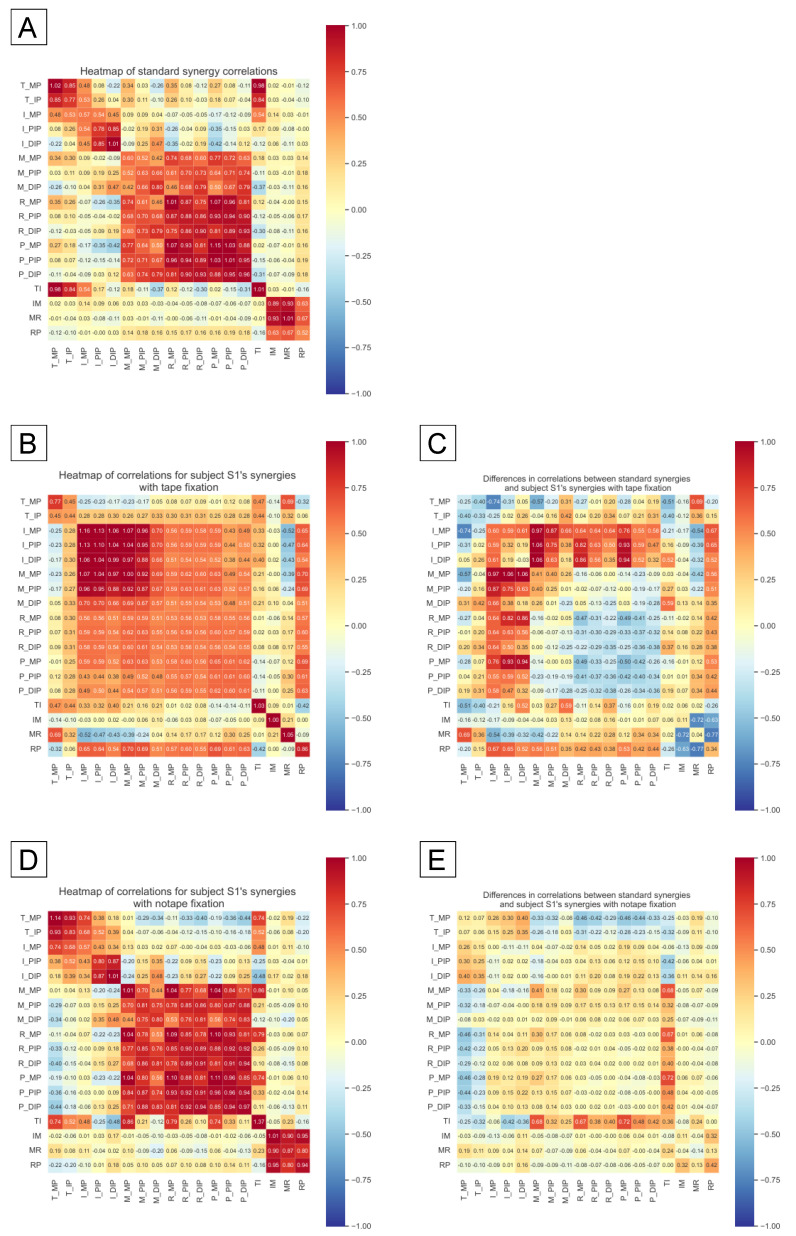
Heatmap showing the correlation between the synergies obtained when subject1 is normal and when the fingers are taped. (**A**) Heatmap of the correlation of standard synergies. (**B**) Heatmap showing the correlation of synergies obtained when the fingers are taped. (**C**) Heatmap showing the difference in correlation between (**A**,**B**,**D**) Heatmap of the correlation of synergies obtained under normal conditions. (**E**) Heatmap showing the difference in correlation between (**A**,**D**).

Correlations were calculated for each subject’s synergy and standard synergy, and the difference between them was taken. Figure 11 shows a heatmap of the correlation for standard synergy (A), the correlation for subject1 synergy when subject1 is normal (D) and when the fingers are taped (B), and the difference in correlation for each of the two conditions. Heatmap (C) shows the difference in correlation between (A) and (B). Heatmap (E) shows the difference in correlation between (A) and (D). In the graph of differences in correlations, it can be read that the range of dark red and blue colors indicating abnormal synergy is widely distributed, especially in the area where the index and middle fingers fixed with tape are involved. In contrast, Heatmap (E) visualizes that the subject’s finger synergy is similar to the standard type by indicating there is no abnormal finger coordination.

### 3.3. Experiment 3

**Figure 12:**
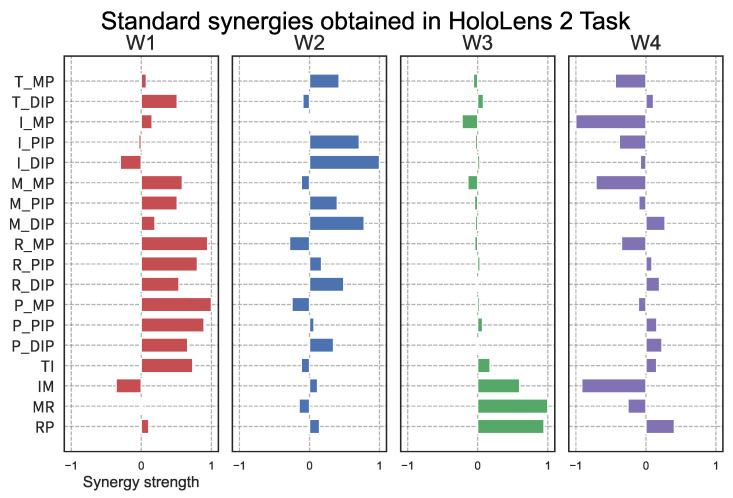
Standard synergy obtained using PCA with varimax rotation from virtual object grasp data obtained in the HoloLens 2 task.

**Figure 13:**
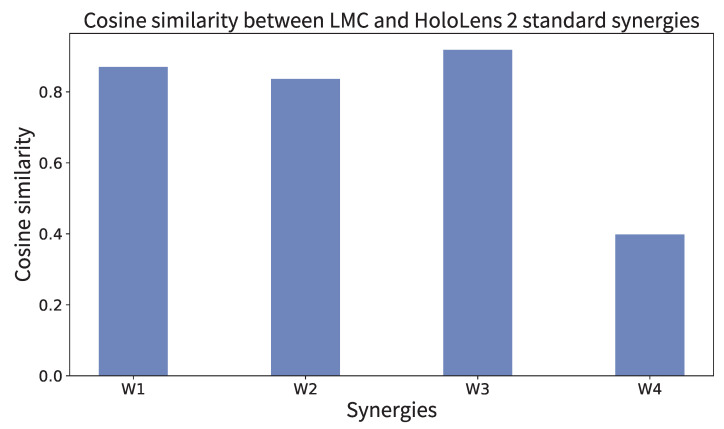
Similarity between the standard synergies obtained in the LMC task and the standard synergies obtained in the HoloLens 2 task.

Figure 12 shows the synergies extracted from the motion data during the HoloLens2 task using PCA with varimax rotation. These synergies were extracted from the data of 8 healthy subjects and represent the top 4 synergies in terms of contribution, as in the LMC task. The similarity between the synergies obtained in the HoloLens2 task and the standard synergies obtained in the LMC task is shown in Figure 13. These results indicate that synergies 1–3 are similar in HoloLens2 as in LMC. On the other hand, synergy 4 had a low similarity. HoloLens2 had a synergy in which many finger joints were coordinated, which may be related to the difference in the hand sensing system between the two systems. However, the principal three synergies could represent the common hand synergy.

The Table 1 summarizes the average time taken to complete the task and the failed attempts for both the LMC task and the HoloLens 2 task. There was little difference in task completion time for both tasks, indicating that hand function assessment can be performed in an MR environment with time efficiency comparable to that of a VR environment. However, the average number of failed attempts was lower for the HoloLens 2 task, suggesting that it may be a more accessible and user-friendly interface, especially by providing better depth perception to the user for the task space.

## 4. Discussion

### 4.1. Sparsity of Synergies Obtained with PCA and Combining PCA and VARIMAX Rotation

In Experiment 1, we extracted spatial synergies of grasping thirty-three different virtual objects using PCA and PCA with varimax rotation. Compared to PCA alone, applying varimax rotation resulted in synergies that required the coordination of fewer joints. We first extracted the spatial synergy shown in Figure 6 (left) using the PCA method. The results indicated that synergy W1, which represents all finger flexion-extension coordination, was obtained. A characteristic of PCA is that the first principal component captures most of the variance, while subsequent components reflect the residual variance. Consequently, W1 encompassed all joint displacements. Additionally, W2 and W3 also yielded synergies that included the coordination of multiple joints, resulting in high densities regarding finger motions.

Figure 6 right shows the synergies extracted using PCA with varimax rotation. Figure 6 suggests that the synergies extracted using varimax rotation are sparser than those extracted using PCA alone. The functions of the four synergies extracted by PCA with varimax rotation are as follows:

- W1: Flexion-extension coordination of the ring and pinky fingers + involuntary movement of middle finger
- W2: Flexion-extension movement of the index finger
- W3: Flexion-extension + adduction-abduction movement of the thumb
- W4: Adduction-abduction coordination of the index, middle, ring, and pinky fingers

The coupling of the ring and pinky fingers, along with the involuntary movement of the middle finger in W1, may be attributed to the tendinous linkage between the middle, ring, and pinky fingers [48]. The synergies in W2 and W3 likely arise from the independent anatomical structures of the index and thumb fingers, respectively, which allow for movement through separate tendons. The coordinated adduction and abduction movements of the four fingers in W4 are thought to be produced by the palmar and dorsal interosseous muscles. Given the biological validity of finger joint coordination movements and the alignment of the human central nervous system with the sparse synergy reported by Prevete et al. [49], we employed PCA and the varimax rotation method as measures of hand function. The result could represent reasonable sparse synergy, which can be easily associated with the motion primitive of finger coordination.

### 4.2. Synergy-Based Detection of Abnormal Hand Movements

Experiment 2 examined the differences between normal hand movements and those with taped fingers. We suggest that hand function assessment, which is challenging from a task performance standpoint, and the hand coordination abnormality may be more effectively evaluated through the lens of synergy. From Figure 9, as no clear trends emerged in the time and success rate scores for the task performance, these metrics were deemed ineffective for evaluating hand function. This may be attributed to the simplicity of the task, which involved grasping a virtual object and moving it; however, the task is based on previously proposed daily tasks. Additionally, the use of other parts of the hand as compensatory strategies during object grasping likely did not lead to variations in the performance metrics. This phenomenon is characteristic of the hand’s multiple degrees of freedom and high redundancy, and human adaptability by exploring the redundant control space. Subsequently, we explored the potential for detecting hand abnormalities through the analysis of synergy. Figure 10 illustrates a comparison of the similarity between the subject’s synergy and the standard synergy, revealing a tendency for higher similarity without tape compared to when tape was applied. In the correlation heat map presented in Figure 11, abnormal synergies were identified in the index and middle fingers during tape fixation, in contrast to standard synergies. The detection of these abnormalities coincided with the pseudo-failure setting (the area where tape was applied), indicating the systematic feasibility of visualizing abnormal joint linkage based on the correlation of synergies.

Here, it should be noted that the current abnormal hand movements are created by taped fingers as a preliminary test with the absence of clinical validation.

### 4.3. Synergy Extraction of Hand Movement Using HoloLens 2

In Experiment 3, synergies obtained from the HoloLens 2 task were compared to those derived from the LMC task. Similar synergies were identified from W1 to W3, while distinct synergies were extracted in W4. This can be attributed to three factors. The first is the change to a format in which the object is grasped from below. It is possible that the subject’s hand movements were influenced by gravity or by grasping from an unfamiliar direction. Second, there is the possibility that the presentation style using MR may have influenced the results. It is because the camera for observing the hand is oriented to view from the bottom in the case of LMC, whereas it is viewed from the head-mounted display in the case of HoloLens. Third, the accuracy of hand recognition played a role. The HoloLens 2 task did not capture the synergy related to thumb movement that was evident in the LMC task, suggesting that the HoloLens 2 may not have recognized the thumb effectively during the grasping motion. These considerations should be taken into account when assessing hand function by using the HoloLens system. However, except for the thumb motion, the hand synergy was well captured even with HoloLens.

A comparison of task completion times revealed little difference between the two tasks. Despite the task variation, MR maintained its time efficiency. Additionally, the number of task failures was lower for the HoloLens 2 task. Two main reasons account for this reduction in failures. First, MR’s spatial presentation of the object may have facilitated a clear understanding of the distance to the target for the user, which is beneficial for HoloLens use. Second, the MR task exhibited a virtual hand superimposed on the subject’s real hand. In the LMC task, subjects must focus on both their own hand and the virtual hand displayed on the screen. Conversely, in MR, they only need to concentrate on one location. These factors suggest that MR may be more suitable for tasks that are easier to perform object manipulation than those using VR with LMC.

## 5. Conclusions

In this study, we developed an automatic finger motor function evaluation system using a virtual object manipulation training game and spatial synergies extracted through principal component analysis (PCA) with varimax rotation. The use of PCA with varimax rotation enabled the extraction of biologically meaningful and sparse synergies specific to virtual object grasping and manipulation, a result that is difficult to achieve with PCA alone.

Furthermore, we demonstrated that it is possible to quantitatively evaluate finger function and detect abnormal areas in terms of synergy, even in daily manipulation tasks where individual finger performance evaluation is difficult and even under the condition that the game performance information does not provide a difference. We also developed an MR task for manipulating a virtual object using HoloLens 2. By utilizing MR technology, participants could better recognize the distance to the virtual object and align their hands with the virtual hand, making the task more user-friendly. The system developed in this study can assess hand motor function, which is challenging to evaluate solely through task performance, across ten different object manipulation tasks. This system is expected to apply to the screening tests for patients with mild motor impairment and also to the recovery process while quantifying the individual synergy structure during motor function improvement after repetitive rehabilitations and facilitating efficient rehabilitation by identifying abnormal areas.

However, several issues remain to be addressed in this study. First, there is a need to increase the number of synergy samples from healthy subjects to establish standard synergies. In this study, we created only one set of standard synergies; however, due to individual differences in the extracted synergies, many healthy subjects exhibit slightly different patterns. Therefore, it is essential to expand the number of subjects to establish the standard synergy used as models. Additionally, clinical experiments with patients who have hand disabilities are necessary for future study. In this study, we simulated hand disabilities using tapes fixed to the fingers, which is a limitation of the current study, with a lack of diversity (e.g., age, gender, handedness). The focus of the current study was to see if synergies can be systematically obtained that are valid in healthy subjects. However, actual patients often present with more complex hand abnormalities that cannot be adequately replicated. Thus, we believe that involving actual patients in testing the game and gathering their feedback will significantly improve the system for the future. Future validation in heterogeneous and patient cohorts is under consideration together with our university hospital, aiming to apply for the rehabilitation evaluation process for patients who have cerebrovascular disease and cognitive impairment.

## Figures and Tables

**Table 1:** Task completion time and number of failures in LMC task and HoloLens 2 task.

	Task Completion Time [s]	Failed Attempts
LMC task	223.5±80.0	10.6±6.7
HoloLens 2 task	227.4±68.6	6.9±3.7

## Data Availability

The data that support the findings of this study are available from the corresponding author upon reasonable request.

## Supplementary Materials

The following supporting information can be downloaded at: https://www.mdpi.com/article/10.3390/s25072080/s1, Movie S1. A scene for Leap Motion object manipulation game with VR. Movie S2. Scene of HoloLens 2 object manipulation game with MR. Movie S3. Animation of the extracted finger motion synergies by PCA and PCA+Varimax.
